# Supplementary material for: Worry about Radiation and Its Risk Factors Five to Ten Years after the Fukushima Nuclear Power Plant Disaster
Source: Int J Environ Res Public Health. 2022 Dec 16;19(24):16943. doi: 10.3390/ijerph192416943 (PMC9778659; doi:10.3390/ijerph192416943)
Supplement: Supplementary file 1 [file ijerph-19-16943-s001.zip › ijerph-2051603-supplementary.pdf]

Supplement

Supplemental Table S1. Comparisons of the level of worry about radiation between the respondents to the next survey and those who dropped out or whose score was missing in each survey.

	Respondents [1)]	Worry about radiation at the preceding survey		
	N	Mean	SD	P [2)]
		Worry about radiation at T1		
T2 respondents	1,158	14.9	4.4	0.945
Drop out or missing	596	14.9	4.5	
		Worry about radiation at T2		
T3 respondents	833	14.2	4.2	0.069
Drop out or missing	382	13.7	4.2	
		Worry about radiation at T3		
T4 respondents	740	14.2	4.5	0.822
Drop out or missing	156	14.1	4.3	
		Worry about radiation at T4		
T5 respondents	678	13.6	4.5	0.503
Drop out or missing	92	14.0	4.4	

SD, standard deviation

1) Number of respondents to each survey for whom the score of worry about radiation in the preceding survey was not missing.

2) The scores of respondents and of those who dropped out or whose score was missing were compared using a t-test.

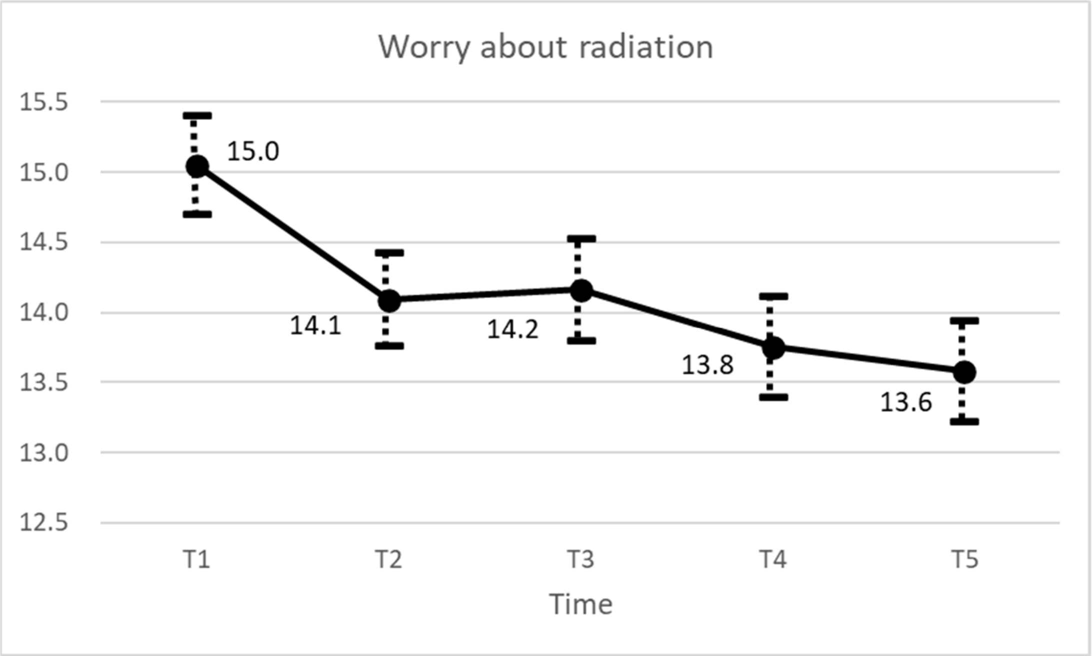

Supplemental Figure S1. Time course change in worry about radiation (means and 95% confidence intervals) five-to-ten years after the nuclear power plant accident among those who responded to all the surveys (N=607). T1: baseline survey or five years after the nuclear power plant accident; T2: second survey or 20 months after T1; T3: third survey or 32 months after T1; T4: fourth survey or 44 months after T1; T5: fifth survey or 56 months after T1. The differences between the scores at the five time points were statistically significant (repeated-measures ANOVA; $F=25.67$, $df=4, 2424$, $p<0.001$).
